# Detection of changes in mitochondrial hydrogen sulfide *in vivo* in the fish model *Poecilia mexicana* (Poeciliidae)

**DOI:** 10.1242/bio.041467

**Published:** 2019-05-15

**Authors:** Gigi Y. Lau, Nicholas Barts, Richard C. Hartley, Michael Tobler, Jeffrey G. Richards, Michael P. Murphy, Sabine Arndt

**Affiliations:** 1 Department of Zoology, University of British Columbia, 6270 University Boulevard, Vancouver, BC V6T 1Z4, Canada; 2 Division of Biology, Kansas State University, 116 Ackert Hall, Manhattan, KS 66506, USA; 3 WestCHEM School of Chemistry, University of Glasgow, Glasgow G12 8QQ, UK; 4 MRC Mitochondrial Biology Unit, University of Cambridge, Hills Road, Cambridge CB2 0XY, UK; 5 Institute for Immunology, University Medical Center of the Johannes Gutenberg University Mainz, Langenbeckstr. 1, Mainz 55131, Germany

**Keywords:** MitoA, Fish, Hydrogen sulfide, Mass spectrometry probe, Mitochondria

## Abstract

In this paper, we outline the use of a mitochondria-targeted ratiometric mass spectrometry probe, MitoA, to detect *in vivo* changes in mitochondrial hydrogen sulfide (H_2_S) in *Poecilia mexicana* (family Poeciliidae). MitoA is introduced via intraperitoneal injection into the animal and is taken up by mitochondria, where it reacts with H_2_S to form the product MitoN. The MitoN/MitoA ratio can be used to assess relative changes in the amounts of mitochondrial H_2_S produced over time. We describe the use of MitoA in the fish species *P. mexicana* to illustrate the steps for adopting the use of MitoA in a new organism, including extraction and purification of MitoA and MitoN from tissues followed by tandem mass spectrometry. In this proof-of-concept study we exposed H_2_S tolerant *P. mexicana* to 59 µM free H_2_S for 5 h, which resulted in increased MitoN/MitoA in brain and gills, but not in liver or muscle, demonstrating increased mitochondrial H_2_S levels in select tissues following whole-animal H_2_S exposure. This is the first time that accumulation of H_2_S has been observed *in vivo* during whole-animal exposure to free H_2_S using MitoA.

This article has an associated First Person interview with the first author of the paper.

## Abstract (precis)

**Summary:** We established the mitochondria-targeted mass spectrometry probe MitoA for H_2_S detection *in vivo* in the animal model *Poecilia mexicana* for the first time.

## INTRODUCTION

Before the rise in atmospheric oxygen (O_2_) levels ∼800 million years ago, oceans were euxinic; that is, both anoxic and sulfidic. The rise in O_2_ was associated with an increased abundance of cyanobacteria and plants that are thought to have eliminated sulphide, which was an abundant energy source (Olson and Straub, 2016). Eventually, organisms adapted mechanisms that used O_2_ to harvest energy and support cellular processes. However, hydrogen sulfide (H_2_S) still retained an important role in cellular function, serving as an important signaling molecule. Indeed, the pathway for H_2_S regulation is highly conserved across modern taxa (Olson and Straub, 2016).

Modern environments that have high H_2_S levels are primarily limited to deep-sea hydrothermal vents (Olson and Straub, 2016), where levels can be 3–110 mmol kg^−1^. Cold seeps and freshwater springs and caves fed by H_2_S-enriched groundwater have been found with levels of H_2_S up to 1200 µM (Tobler et al., 2011). These habitats are characterized by low biodiversity due to the sensitivity of modern metazoans to environmental H_2_S, with toxic levels between ∼20 and 40 µM (Bouillaud and Blachier, 2011; Olson and Straub, 2016). The primary reason for H_2_S toxicity is due to the ability of H_2_S to inhibit the respiratory enzyme cytochrome *c* oxidase, which hinders the ability of mitochondria to generate ATP aerobically (Petersen, 1977). Yet, endogenous H_2_S is also produced enzymatically in the nM range in the cytosol and mitochondria (Olson and Straub, 2016) and has important signaling roles, for example, in the regulation of vascular tone (Dombkowski et al., 2004; Olson et al., 2006), metabolic rate suppression (Revsbech et al., 2014) and osmoregulation (Kumai et al., 2015). Deviations from endogenous H_2_S homeostasis have been linked to a number of pathologies in mammalian models, such as cardiovascular disease (e.g. myocardial infarct; Arndt et al., 2017; Elrod et al., 2007; Johansen et al., 2006), neurodegenerative diseases (Kimura and Kimura, 2004) and inflammation (Zanardo et al., 2006). As H_2_S oxidation occurs exclusively in mitochondria, these organelles thus play key roles in maintaining cellular H_2_S homeostasis. Studies on the role of H_2_S within mitochondria are therefore integral in enhancing our understanding of the physiological and pathophysiological role of this signaling molecule and how it influences whole-animal performance. To investigate this relationship, a sensitive and reliable method of monitoring changes in intracellular H_2_S *in vivo* is required.

There are numerous H_2_S detection methods available, including the use of fluorophores, specific enzyme-linked assays (Cline, 1969; Takano et al., 2016) and gas chromatography (Furne et al., 2008). However, these detection methods all come with limitations, namely low sensitivity, and more importantly, they cannot be used reliably in living organisms (Olson et al., 2014). Arndt et al. (2017) developed and characterized a mitochondria-targeted ratiometric mass spectrometry probe called MitoA, which is a triphenylphosphonium cation (TPP^+^) conjugated to an aryl azido group, to detect H_2_S *in vivo* and applied it to murine models. This probe was shown to rapidly accumulate within mitochondria, due to its TPP^+^ moiety and, upon reacting with H_2_S, formed the stable product, MitoN, named after its aryl amine group. The precursor MitoA and product MitoN can then be extracted from sampled tissue and analyzed via tandem mass spectrometry (LC-MS/MS). This approach allows for the sensitive detection of MitoA and MitoN, enabling the analysis of changes in *in vitro* and *in vivo* H_2_S level in different tissues using the ratio of MitoN to MitoA (MitoN/MitoA).

So far, *in vivo* H_2_S levels have predominantly been investigated in murine models that do not encounter high H_2_S in their environment and also cannot tolerate high environmental H_2_S levels. We thus aimed to test whether MitoA can be used in animal models that naturally inhabit H_2_S-rich environments to ultimately study adaptive mechanisms underlying H_2_S tolerance. *Poecilia mexicana* is a widespread fish within the family Poeciliidae that inhabits environments across the Atlantic coast of Mexico and parts of Central America. Multiple lineages of this species have independently colonized habitats rich in H_2_S in southern Mexico (Tobler et al., 2011). Sulfide-tolerant populations are locally adapted and exhibit genetic, physiological, behavioral and morphological differences from nearby sulfide-intolerant populations (Tobler et al., 2018). The ability of certain populations of *P. mexicana* to tolerate high levels of environmental sulfide could be, in part, due to cytochrome *c* oxidase being less susceptible to H_2_S as it was shown for other fish (Pfenninger et al., 2014) and modifications of genes in detoxification pathways (Kelley et al., 2016; Zhang et al., 2017). However, the amount of contribution of those adjustments remains unclear, i.e. whether the H_2_S tolerance in *P. mexicana* results predominantly from efficient H_2_S oxidation that keeps the overall *in vivo* H_2_S levels low or if the *in vivo* H_2_S levels increase in the whole organism, which might indicate less H_2_S susceptible enzymes. Using laboratory-bred populations of *P. mexicana* that originated from a H_2_S-rich spring, we here describe how to establish the use of MitoA in a new species for a time course study. Then, in a proof-of-concept study, we exposed captive-bred *P. mexicana* to high H_2_S treatment and determined changes in tissue levels of MitoN/MitoA.

## RESULTS AND DISCUSSION

Our optimization and proof-of-principle studies showed that MitoA could be used in *P. mexicana* exposed to high environmental H_2_S to detect *in vivo* changes in mitochondrial H_2_S.

### Part 1: optimizing use of MitoA in *P. mexicana*

**Fig. 1.**
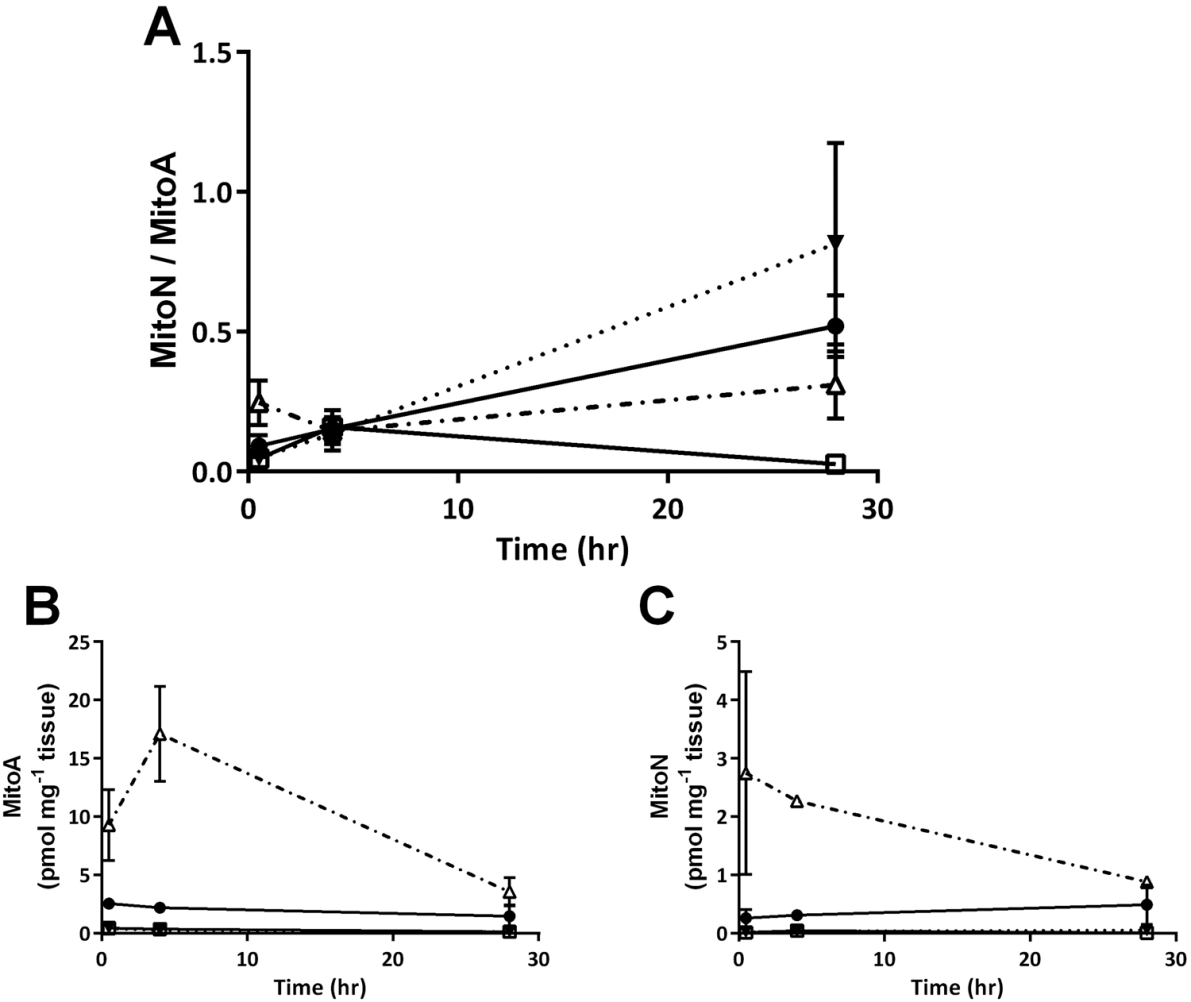
Time course of (A) MitoN/MitoA, (B) MitoA, and (C) MitoN of *P. mexicana* after intraperitoneal MitoA injection. Liver (hollow triangle), gills (solid circle), muscle (solid inverted triangle) and brain (hollow square) were sampled at 0.5, 4 and 28 h. Data presented are means±s.e.m., *n*=3.

The goal of this initial 28 h time course experiment was to determine if *P. mexicana* tissues showed MitoA uptake and also whether the probe would be retained for H_2_S exposure experiments of 4 h duration. Our results show that levels of both MitoA and MitoN were detectable 0.5 h after injection (Fig. 1B,C), resulting in a detectable MitoN/MitoA (Fig. 1A) up to 28 h post-injection. Furthermore, MitoA showed tissue-specific uptake, which was highest in gill and liver and lowest in muscle and brain (Fig. 1B), but all at detectable levels 28 h after the injection. Specifically, brain MitoN concentration was increased at 4 h compared to levels at 0.5 h but decreased back to starting levels at 28 h. This, however, was not reflected in any significant changes in MitoN/MitoA (*P*=0.139 and *P*=0.421; Table 1). Gill MitoN/MitoA significantly increased after 28 h compared to 0.5 h, indicating an increase in mitochondria H_2_S in gill mitochondria (*P*=0.021; Table 1). This highlights that the best time point in experiments depends on the tissue of interest and should be optimized for every new animal model. There were no other changes in concentrations of MitoA, MitoN and MitoN/MitoA over the time course examined. Interestingly, MitoA injected via tail vein into mice did not show significant uptake into the brain (0.001 pmol/mg tissue; Arndt et al., 2017). Thus, the measurable levels of MitoA in *P. mexicana* brains (0.43 pmol/mg tissue) suggest that the blood-brain barrier in this species may be more permeable to MitoA than that in mice and that MitoN/MitoA can be obtained from brains of *P. mexicana*.

**Table 1.**
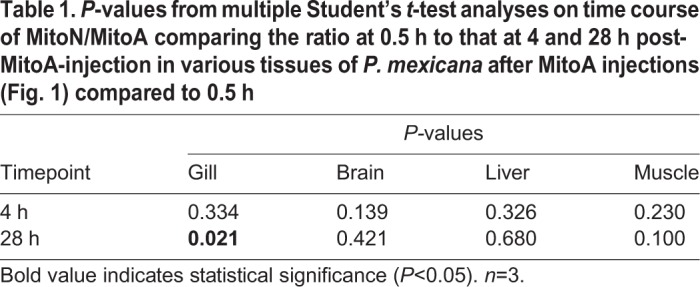
***P*-values from multiple Student's *t*-test analyses on time course of MitoN/MitoA comparing the ratio at 0.5 h to that at 4 and 28 h post-MitoA-injection in various tissues of *P. mexicana* after MitoA injections (****Fig. 1****) compared to 0.5 h**

### Part 2: response of MitoA in *P. mexicana* to H_2_S exposure

Our H_2_S exposure of 59 µM is a concentration that *P. mexicana* routinely experience in their natural habitat (Tobler et al., 2006) and causes high levels of mortality in non-tolerant populations (Plath et al., 2013). There was a significant increase in MitoN/MitoA in the gill (*P*=0.033) and brain tissues (*P*=0.049). Although liver and muscle appeared to show a general trend of an increase in MitoN/MitoA after 5 h exposure to high H_2_S, these were not significantly different (*P*=0.15 and *P*=0.083, respectively). These results indicate that whole-animal H_2_S exposure only increased mitochondrial H_2_S levels in select tissues.

**Fig. 2.**
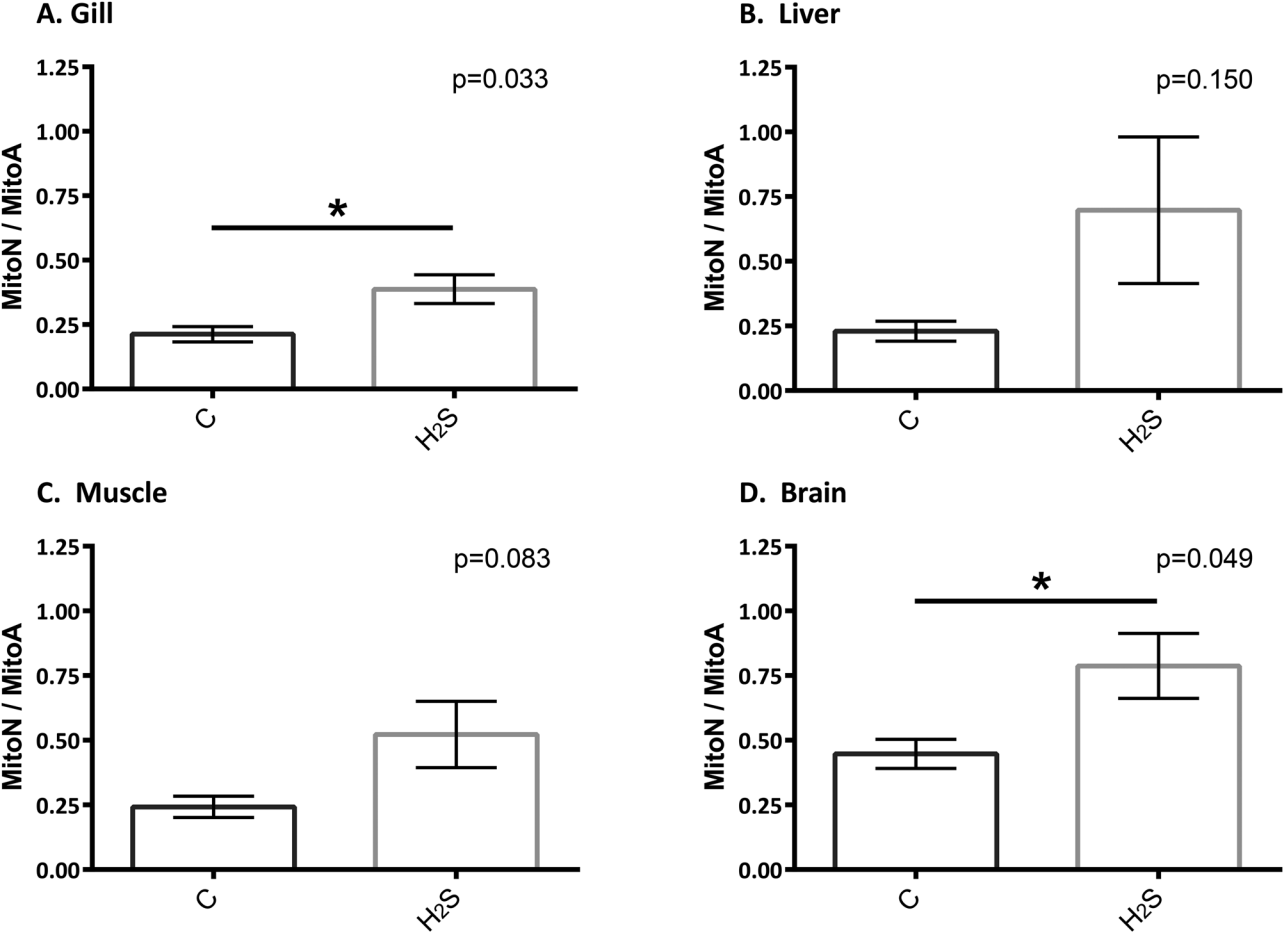
MitoN/MitoA ratio in *P. mexicana* (A) gill, (B) liver, (C) muscle and (D) brain after 5 h control (represented by ‘C’) or H_2_S exposure (59 µM). Samples were tested for significance using an unpaired, two-tailed *t*-test. Data presented are means±s.e.m., *n*=4.

Up until this present study, MitoA has not been used to monitor changes in *in vivo* H_2_S in a study animal that has been exposed to high environmental H_2_S. Arndt et al. (2017) demonstrated that changes in H_2_S levels *in vivo* were detectable using MitoA, showing that the probe is sensitive enough to monitor physiologically relevant H_2_S levels. Although we opted to study a captive-bred population of *P. mexicana* that were naïve to H_2_S, they were still able to tolerate exposure to a high concentration of H_2_S. The MitoN/MitoA increase was relatively subtle, possibly due to adaptive mechanism of sulfide tolerance this population possesses. A previous study has shown that H_2_S-tolerant population of *P. mexicana* showed constitutive upregulation of genes involved in sulfide–quinone reductase (SQR) pathway (Passow et al., 2017), indicating a greater capacity to metabolize H_2_S compared to the non-tolerant population. Our results show that H_2_S accumulated in the gills and the brain in a H_2_S-tolerant species, suggesting that the high environmental H_2_S exceeded the capacity of the gills to breakdown H_2_S and caused build up of H_2_S in other organs (Fig. 2). However, the differences in tissue responses in changes in MitoN/MitoA possibly indicate that there are differences in the ability of the tissue types investigated to metabolize H_2_S. It is also possible that endogenous H_2_S production may have been affected by environmental H_2_S exposure. Though there is little evidence for altered gene expression in these pathways in sulfur-tolerant *P. mexicana* (Kelley et al., 2016), whether there are functional differences at the protein level that is associated with these tissue specific responses remains to be determined.

### Part 3: additional notes for MitoN/MitoA analysis for whole animal exposure experiments

The MitoA and MitoN amount in tissue varies depending on the tissue-specific uptake of MitoA due to variation in mitochondrial volume density and the MitoA amount injected per fish weight. However, it must be noted that neither factors ultimately impact the assessment of the MitoN/MitoA ratio used to indicate changes in mitochondrial H_2_S levels (Arndt et al., 2017), as long as the amount of neither MitoA injected or the product MitoN generated are below the level of detection of MS.

Additionally, though we show here the effect of whole-animal H_2_S exposure on MitoN/MitoA in different tissue types, one must be careful making comparisons between tissues. This is because the accumulation of H_2_S in different tissues may vary and that the baseline MitoN/MitoA ratios vary depending on tissue, i.e. the absolute MitoN/MitoA ratios between e.g. liver and brain should not be compared.

### Summary

The ability to measure endogenous H_2_S in whole-animal experiments can help to address long-standing questions of H_2_S physiology. Using comparative animal models, it is possible to elucidate the mechanisms of H_2_S tolerance, which could be due to improved regulation (i.e. increased breakdown of H_2_S in tolerant populations compared to non-tolerant populations) or resistance (i.e. H_2_S increases in both ecotypes, but this only impacts the performance of non-tolerant populations rather than the tolerant populations) or the role of H_2_S in ischemia-related pathologies. In this study, we have outlined the use of MitoA in a teleost fish and demonstrated that it is a powerful tool to evaluate changes in mitochondrial H_2_S *in vivo* and elucidate physiological mechanisms of H_2_S metabolism and tolerance.

## MATERIALS AND METHODS

### Equipment and chemicals

The MitoA-related compounds (MitoA, MitoN, d_15_-MitoA, d_15_-MitoN) were synthesized as described previously (Arndt et al., 2017). MitoA is commercially available from Cayman Chemicals (https://www.caymanchem.com/product/22702).

### Animal care

All fish used in the study were captive-bred *P. mexicana* from stocks originally collected from the El Azufre I spring in the Tacotalpa River drainage in Mexico (Tobler et al., 2011). Fish used for part 1 were held at the University of British Columbia and those used for part 2 were held at the Kansas State University. *P. mexicana* were maintained in aquaria filled with artificial water (0.6 mM CaSO_4_·2H_2_O, 0.3 mM CaCl_2_, 0.5 mM NaHCO_3_, 0.05 mM KHCO_3_, 0.2 mM MgSO_4_·7H_2_O) in environmental chambers and kept at 25°C. All fish were fed *ad libitum* three times a week with commercial feed (Purina Aquamax) and fasted 24 h prior to experiments. All experimental procedures were reviewed and approved by The University of British Columbia Animal Care Committee (A13-0309) and the Kansas State University Institutional Animal Care and Use Committee (#3473).

### Preparation of MitoA for intraperitoneal (IP) injection

The same injection and sampling procedures were used for both parts of this study. A 10 mM MitoA stock (in ethanol) was prepared and stored in the dark at −20°C. On the day of the experiment, the MitoA stock was diluted in phosphate buffered saline (PBS from Sigma-Aldrich) for injection. For each fish, a total volume of 50 µl containing 8 nmol MitoA was prepared [with a small amount of food coloring (Club House from McCormick Canada) added to facilitate visibility].

### Part 1: determining tissue MitoA and MitoN changes over 28 h (no H_2_S exposure)

Previous characterization of MitoA used in mice showed that the probe was taken up into tissues within 1 min following intravenous injection and was excreted at a steady rate (Arndt et al., 2017). Thus, the sensitivity of the probe diminishes over time. To determine the time course of MitoA uptake following IP injection in fish and the functional period over which experiments can be conducted in *P. mexicana*, individual fish were weighed (1.46±0.24 g, *n*=8), quickly placed on a wet paper towel, injected with MitoA using a BD Ultrafine II syringe via IP injection (3/10 ml cc, 31G) and placed into a recovery container (whole process was completed in under 10 s). Injected fish were then placed into aerated water of the same chemistry and temperature (25°C) as their holding conditions to recover. The aquaria had a filter and an air pump to ensure sufficient mixing and aeration. At 0.5, 4 and 28 h after injection of MitoA, individual fish were anaesthetized with benzocaine (0.5 g/l), and brain, liver, gill and muscle samples were extracted and frozen in liquid nitrogen. Samples were stored at −80°C until further analysis.

### Part 2: effects of 5 h H_2_S exposure on MitoN/MitoA

The stock fish were born and raised under common garden conditions at Kansas State University and had never been exposed to H_2_S. H_2_S exposure methods were modified from a previous study (Tobler et al., 2014). Fish were weighed (0.82±0.086 g, *n*=8) and then anaesthetized in a buffered MS-222 solution (85 mg l^−1^; Lumb, 1963), restrained on a wet paper towel, injected with MitoA, and subsequently individually placed in 1 l containers with 250 ml of aerated water of the same chemistry and temperature as acclimation conditions within a temperature-controlled (25°C) water bath. After acclimating for 1 h, the containers were sealed, and peristaltic pumps were used to continuously supply either an H_2_S solution or non-sulfidic control from reservoirs at a flow rate of 150 ml h^−1^ for 5 h. Due to the excretion of both MitoA and MitoN over time, paired controls not exposed to H_2_S were sampled at the same time and were essential to serve as points of comparison. H_2_S stock solutions were prepared by dissolving 2.4 g sodium sulfide nonahydrate (Na_2_S·9H_2_O) in 2 l deoxygenated water under anoxic conditions in a glovebox. H_2_S concentrations were determined using a methylene blue assay test kit (Hach Company, Loveland, Colorado) with final H_2_S concentrations measuring at 59±9 µM, whereas controls were below the detection limit. Control solutions lacked sodium sulfide. At the end of the exposure, fish were euthanized by pithing, and brain, liver, gill and muscle samples were extracted, frozen in liquid nitrogen, and stored at −80°C until further analysis.

### Tissue extraction of MitoA and MitoN

The protocol for extraction of MitoA and MitoN is based on that of Arndt et al. (2017) for mammalian tissue with some differences outlined below for *P. mexicana*. Frozen tissues were first weighed into chilled 2 ml Eppendorf tubes (in mg in part 1: gill 60±10, liver 21±3, muscle 93±10, brain 17±2.0; in part 2: gill 16±2.3, liver 12±1.7, muscle 35±6.1, brain 9.8±0.72). If available, more tissue (up to 100 mg) can be used for tissues that show lower uptake of MitoA to increase sensitivity. 210 µl 60% acetonitrile spiked with internal standards (100 pmol d_15_-MitoA and 100 pmol of d_15_-MitoN) was added to each sample. All chemicals for extraction and mass spectrometry analysis were of HPLC grade. *P. mexicana* tissue was homogenized using a Bullet Blender (Storm24; Next Advance) in 95% acetonitrile, which was used for mouse samples (Arndt et al., 2017), but yielded a viscous homogenate that was difficult to process (G.Y.L., personal observation). Instead, *P. mexicana* samples were homogenized using ∼50 mg beads (0.5 mm diameter zirconium oxide beads; Next Advance) with a Bullet Blender for 3 min at speed 8. Gill samples were homogenized for an extra minute at speed 9. The samples were then centrifuged at 16,000 ***g*** for 10 min after which the supernatant was transferred to a new tube. The tissue and beads were re-homogenized with another 200 µl 60% acetonitrile and re-centrifuged, the supernatants were combined and allowed to stand for 30 min at 4°C to precipitate proteins. The samples were then centrifuged at 16,000 ***g*** for 10 min to pellet the precipitates. The supernatants were loaded onto 96-well Millipore Multiscreen filter plates with low protein binding Durapore membranes (0.45 µm pore size) and centrifuged for 10 min at 1108 ***g***. The filtrate was then transferred into fresh Eppendorf tubes and dried using a speed vacuum centrifuge (CentriVap benchtop vacuum concentrator, Labconco). Samples were stored dried at 20°C until analysis by LC-MS/MS. To prepare for LC-MS/MS analysis, the samples were resuspended in 20% acetonitrile with 0.1% formic acid, centrifuged for 10 min at 16,000 ***g*** and transferred to mass spectrometry vials (TrueView™ LCMS Certified, Waters).

### LC-MS/MS analysis

For LC-MS/MS analyses, the mass spectrometer in positive ion mode was connected in series to an I-class Aquity LC system (Waters). Nitrogen and argon were used as curtain and collision gases, respectively. Samples were stored in an autosampler at 4°C and 2 µl was taken into a 15 µl flow-through needle and RP-UPLC at 40°C using an Acquity UPLC^®^ BEH C18 column (1×50 mm, 1.7 µm; Waters) with a Waters UPLC filter (0.2 µm). MS buffers A (95% water/5% ACN/0.1% FA) and B (90% ACN/10% water/0.1% FA) were infused at 200 µl/min using the following gradient: 0–0.3 min, 5% B; 0.3–3 min, 5%–100% B; 3–4 min, 100% B, 4.0–4.10, 100%–5% B; 4.10–4.60 min, 5% B. Eluant was diverted to waste from 0–1 min and from 4–4.6 min. Compounds were detected in multiple reactions monitoring in positive ion mode. Under these conditions MitoA underwent neutral loss of N_2_ to a nitrene which was used as the parent ion. For quantification, the following transitions were used: MitoA, 437>183; d_15_-MitoA, 452>191; MitoN, 439>183; d_15_-MitoN, 454>191. Standard curves with known amounts of MitoA and MitoN were prepared, spiked with internal standards and extracted following the protocol outlined above (Fig. S1). The peak area of MitoA, MitoN and internal standards of samples and standard curves were quantified using the MassLynx 4.1 software.

### Statistical analyses

Multiple Student's *t*-tests were used to compare time points at 4 h and 28 h to time point 0.5 h (Fig. 1) and Student's *t*-tests when comparing MitoN/MitoA between control and H_2_S exposed fish for each individual tissue (Fig. 2).

## Supplementary Material

Supplementary information
