# Supplementary figures and images for: Detection of changes in mitochondrial hydrogen sulfide in vivo in the fish model Poecilia mexicana (Poeciliidae)
Source: Biol Open. 2019 May 15;8(5):bio041467. doi: 10.1242/bio.041467 (PMC6550084; doi:10.1242/bio.041467)

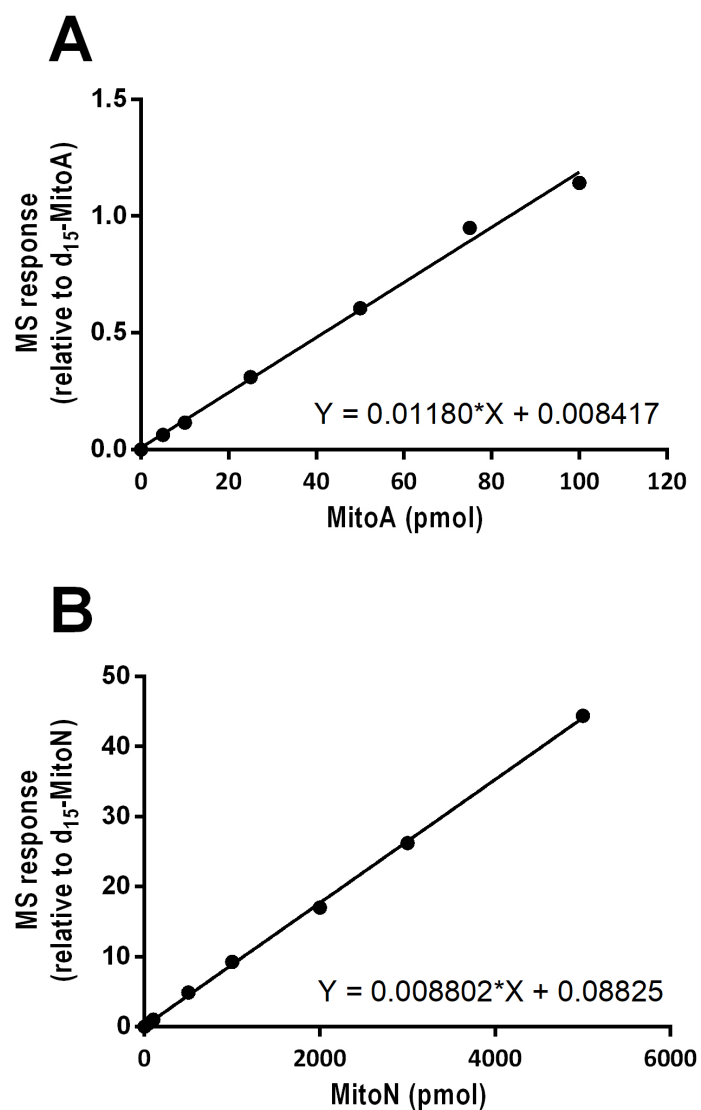

**Fig. S1. Standard curves for (A) MitoA and (B) MitoN for fish tissue**

Supplement: Supplementary information [file biolopen-8-041467-s1.pdf]
